# Inosine and D-Mannose Secreted by Drug-Resistant *Klebsiella pneumoniae* Affect Viability of Lung Epithelial Cells

**DOI:** 10.3390/molecules27092994

**Published:** 2022-05-06

**Authors:** Yuhan Zhang, Ziwei Zhou, Wenxuan Xiao, Yuting Tang, Wei Guan, Jiang Wang, Farui Shu, Jiaqi Shen, Shaoyan Gu, Lu Zhang, Qingzhong Wang, Lixin Xie

**Affiliations:** 1 College of Pulmonary and Critical Care Medicine, Chinese People’s Liberation Army General Hospital, Beijing 100853, China; zhangyuhannku@foxmail.com (Y.Z.); guanwei91@126.com (W.G.); albert_19891117@163.com (J.W.); shaoyan.gu@outlook.com (S.G.); 2 Medical School of Chinese People’s Liberation Army, Beijing 100853, China; 3 State Key Laboratory of Genetic Engineering, Institute of Genetics, School of Life Science, Fudan University, Shanghai 200437, China; zhou_ideal@163.com (Z.Z.); xiao-xwx@foxmail.com (W.X.); 18321656189@163.com (Y.T.); 16307110154@fudan.edu.cn (F.S.); 18307110040@fudan.edu.cn (J.S.); zhanglu407@fudan.edu.cn (L.Z.); 4 Shanghai Centre for Clinical Laboratory, Shanghai 200126, China

**Keywords:** *Klebsiella pneumoniae*, drug resistance, A549, metabolomics, inosine, D-mannose

## Abstract

The antibiotic resistance rates of *Klebsiella pneumoniae* have been steadily increasing in recent years. Nevertheless, the metabolic features of the drug-resistant *Klebsiella pneumoniae* and its associated benefits for bacterial pathogenicity are far from expounded. This study aims to unravel the unique physiological and metabolic properties specific to drug-resistant *K. pneumoniae*. Using scanning electron microscopy (SEM), we observed a thicker extracellular mucus layer around a drug-resistant *K. pneumonia* strain (Kp-R) than a drug-sensitive *K. pneumonia* strain (Kp-S). Kp-R also produced more capsular polysaccharide (CPS) and biofilm, and appeared to have a significant competitive advantage when co-cultured with Kp-S. Moreover, Kp-R was easier to adhere to and invade A549 epithelial cells than Kp-S but caused less cell-viability damage according to cell counting kit-8 (CCK-8) tests. Immunofluorescence revealed that both Kp-R and Kp-S infection destroyed the tight junctions and F-actin of epithelial cells, while the damage caused by Kp-S was more severe than Kp-R. We detected the extracellular metabolites secreted by the two strains with UHPLC-Q-TOF MS to explore the critical secretion products. We identified 16 predominant compounds that were differentially expressed. Among them, inosine increased the viability of epithelial cells in a dose-dependent manner, and an A_2A_R antagonist can abolish such enhancement. D-mannose, which was secreted less in Kp-R, inhibited the viability of A549 cells in the range of low doses. These findings provide potential targets and research strategies for preventing and treating drug-resistant *K. pneumoniae* infections.

## 1. Introduction

*Klebsiella pneumoniae*, a member of the *Enterobacteriaceae* family, is ubiquitous in the environment and exists in the gastrointestinal tracts of both healthy humans and animals [1,2]. Despite existing as a commensal organism, it can cause severe nosocomial and community-acquired infections, which may develop into necrotizing pneumonia, pyogenic liver abscesses and endogenous endophthalmitis, and even endocarditis, septicemia, or other lethal infections [3,4].

Antimicrobial resistance is becoming one of the most severe threats to public health worldwide. The rates of antibiotic resistance among *K. pneumoniae* has continued to grow in recent years [5,6]. Wyres et al. reported that the overall prevalence of extended-spectrum beta-lactamase (ESBL) genes among *K. pneumoniae* isolates from seven major medical institutions across South and Southeast Asia was 47% [7]. Under antibiotic selective pressure, *K. pneumoniae* accumulated inheritable antimicrobial-resistance (AMR) genes primarily via horizontal gene transfer (HGT) from other pathogens [8]. Simultaneously, *K. pneumoniae* can gain stronger intrinsic drug resistance through its specific outer-membrane protein [9], polysaccharides, or biofilms [10], which are relatively impermeable to most hydrophilic and lipophilic drugs [11,12,13,14]. 

Recent studies have uncovered the close association between bacterial metabolism and antibiotic resistance, which may provide a novel perspective on the development of antibiotics [15,16]. Metabolic adaption mechanisms enable pathogens to survive in host immune and antibiotic treatment by altering metabolic pathways. MS-based metabolomics has proved to be a powerful tool in biomarker discovery and drug screening [15]. Shon and his colleagues found that iron-acquisition systems (aerobactin and yersiniabactin) and catabolism of allantoin in *K. pneumoniae* showed an association with hypervirulent pathogenicity [17]. Foschi et al. compared the metabolic profiles of carbapenemase-positive and carbapenemase-negative *K. pneumoniae* strains and identified five differentially expressed metabolites [18]. However, the detailed connections between the antimicrobial-resistant phenotype *K. pneumoniae* and its metabolic profiles remain unclear. 

For many respiratory pathogens, including *K. pneumoniae*, the colonization of host tissues is mediated by the initial adherence to epithelial cells, followed by internalization into cells, which represents an early stage of pneumonia [19]. Epithelial cells can express a broad spectrum of receptors, such as toll-like receptors (TLRs), to identify a pathogen-associated molecular pattern (PAMP) and initiate cellular responses, which eventually lead to the clearance of pathogenic microorganisms [20]. Accordingly, we used the epithelial cells to evaluate bacterial virulence and predict its pathogenicity [21].

Here, with a lung-epithelial-cell infection model, we identified and compared the unique metabolic profiles between Kp-R and Kp-S and explored their pathogenic characteristics. Sixteen predominant differentially expressed metabolites were identified, and inosine and D-mannose were found to influence the cell viability. Our investigation on the correlation between bacterial metabolomics and the drug-resistant phenotype provided potential metabolic biomarkers and targets for novel antibiotics. 

## 2. Results

### 2.1. Colony Morphology and Capsular Polysaccharide (CPS) Quantification

The MICs of the selected antibiotics were listed in Table S1 (Supplementary Materials). 

**Figure 1:**
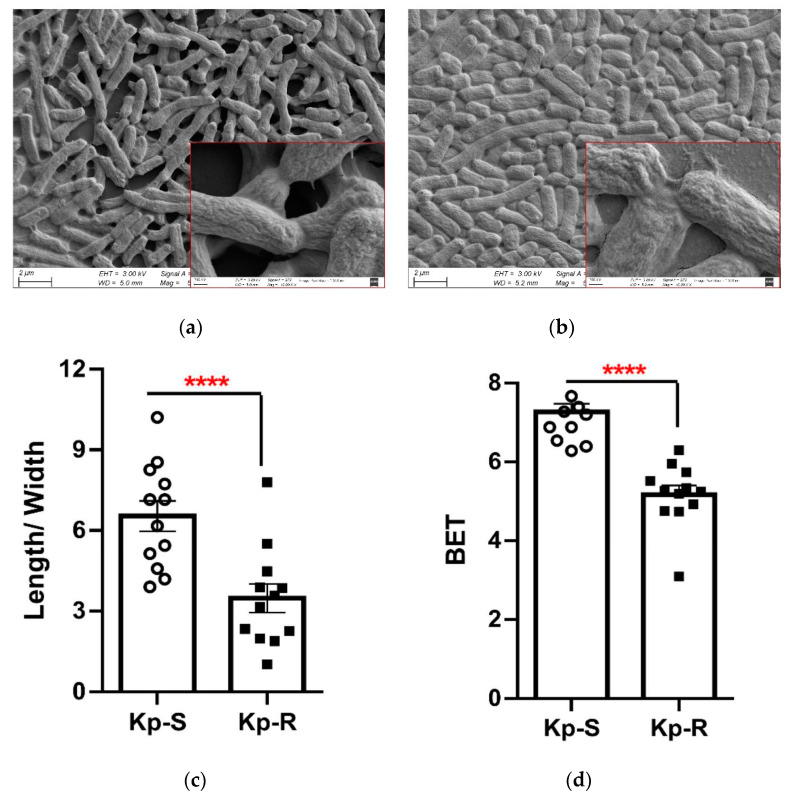

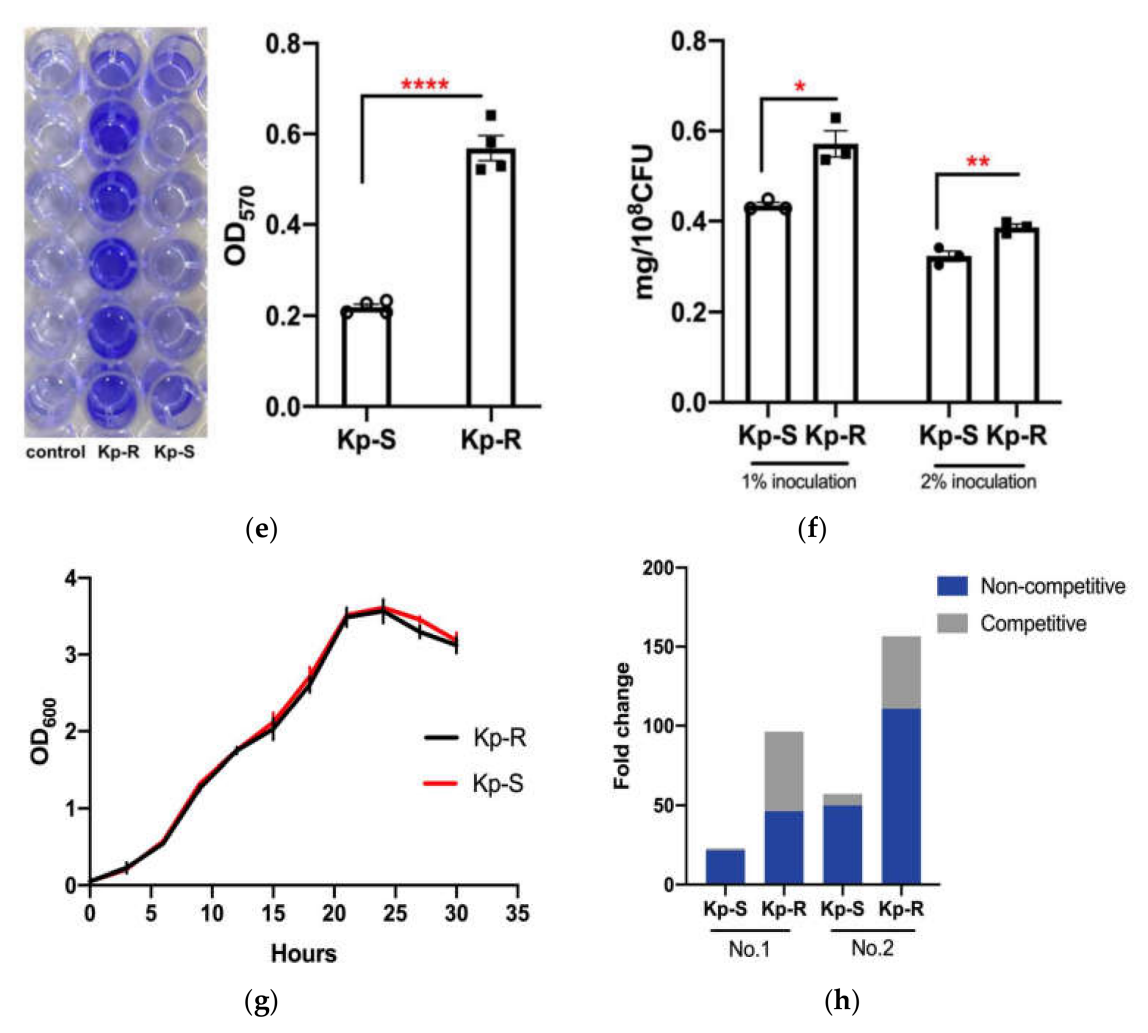
Bacterial morphology, biofilm-formation assay, CPS quantification, growth kinetics and proliferation rates under single or co-culture of Kp-S and Kp-R. (**a**) SEM image of Kp-S. (**b**) SEM image of Kp-R. Images show 4000× and 50,000× magnifications. (**c**) Length–width ratios of the strains. (**d**) BET of the strains. (**e**) Biofilm formation was performed. The biofilms were dyed purple, and in Kp-R’s wells the color was deeper than Kp-S. The histogram showed measurements of OD 570. (**f**) CPS quantification. (**g**) Growth kinetics of Kp-R and Kp-S in the LB medium under shaking culture at 37 °C. (**h**) Proliferation rates under single culture and co-culture. The grey bar represents proliferation rates under single culture (non-competitive condition); the blue bar represents proliferation rates under co-culture (competitive condition). (*, *p* < 0.05, **, *p* < 0.01, ****, *p* < 0.0001. Mean ± SE. Data analyzed by the Student’s *t*-test).

From SEM images, Kp-R appeared to have a thicker extracellular mucus layer (Figure 1b), causing bacterial cells to form linkages with each other, and there was a lack of apparent three-dimensional structure compared to the morphology of Kp-S (Figure 1a). The length–width ratio showed that Kp-R was significantly shorter than Kp-S (length–width ratios were 3.48 ± 1.85 and 6.54 ± 1.96, respectively, shown in Figure 1c). Regarding the specific surface area (BET), Kp-R had a significantly smaller surface area than Kp-S, i.e., 5.17 ± 0.81 and 7.27 ± 0.71, respectively (Figure 1d).

To evaluate the relationship between biofilm-forming ability and the drug-resistant phenotype, we quantified the biofilm formation of Kp-S and Kp-R by staining the strains with crystal violet and measuring OD 570 (Optical density at 570 nm) (Figure 1e). We found that the OD 570 value of Kp-R (0.419 ± 0.06) was significantly higher than Kp-S (0.0695 ± 0.01), confirming that Kp-R produced more biofilm than Kp-S.

Consistently, we found that Kp-R contained more CPS than Kp-S (Figure 1f). With equal amounts of the two strains diluted in concentrations 1% and 2% and cultured overnight, Kp-S formed 0.43 ± 0.01 mg/10^8^ colony-forming units (CFU) CPS while Kp-R formed 0.57 ± 0.05 mg/10^8^ CFU at 1% inoculation volume, and a 2% inoculation volume came to the same conclusion (0.32 ± 0.02 mg/10^8^ CFU and 0.38 ± 0.01 mg/10^8^ CFU, respectively).

### 2.2. In Vitro Growth Characteristics of the K. pneumoniae Strains

We evaluated the growth curve of Kp-R and Kp-S in the Luria-Bertani (LB) medium (Figure 1g) and Brain Heart Infusion (BHI broth) (Figure S1) [22]. There was no significant difference between the two strains under single-culture conditions. Then, we co-cultured them to explore their proliferation under competitive growth conditions. As shown in Figure 1h, after growth overnight with the same initial bacteria amounts, Kp-R proliferated 50 times, and Kp-S proliferated 1.332 times under co-culture conditions. When cultured alone simultaneously, Kp-R proliferated 46.25 times, and Kp-S proliferated 21.5 times. The repeat test confirmed the same results.

### 2.3. Cell Adhesion and Cell Viability of A549 Cell Line Infected with K. pneumoniae 

**Figure 2:**
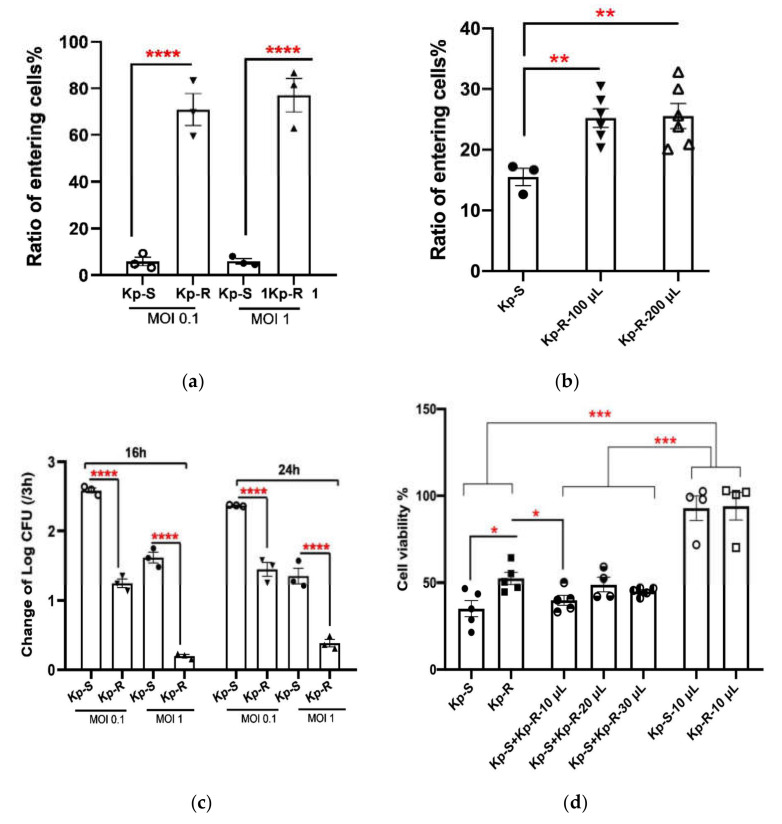
Adherence assay, bacterial intracellular proliferation, and cytotoxicity assay in Kp-R and Kp-S-infected A549 cells. (**a**,**b**) Ratio of entering bacterial cells was enumerated when Kp-R- and Kp-S-infected A549 cells at low infection doses (MOI 0.1 and 1, Figure 2a) and Kp-S (MOI 1) supplemented with supernatants of Kp-R. Kp-R-100 μL showed that the volume of supernatants was 100 μL; Kp-R-200 μL was 200 μL (**b**). (**c**) Intracellular proliferation ability of Kp-R and Kp-S in A549 cells. (**d**) A549 cell viability was evaluated after infected by Kp-S, Kp-R (both MOI 1), Kp-S (MOI 1) supplemented with different volume supernatants of Kp-R and supernatants of Kp-R. *Y*-axis represents cell viability; Kp-R-10 or 20 or 30 μL and Kp-S-10 μL represented appropriate supernatants of Kp-R and Kp-S, respectively. (*, *p* < 0.05, **, *p* < 0.01, ***, *p* < 0.001; ****, *p* < 0.0001). Mean ± SE; Data analyzed by the Student’s *t*-test and one-way ANOVA for multiple groups. The experiments were performed in triplicate.

Lung epithelial cell line A549 was infected with Kp-R and Kp-S to explore the relation between the drug-resistant phenotype and pathogenicity [21]. The results showed that under a low infection dose (multiplicity of infection, MOI 0.1 and 1), the efficiency of bacterial entry into A549 cells was significantly higher in the Kp-R groups (Figure 2a).

Considering that Kp-R showed the remarkable advantage of competitive growth when co-cultured, we wonder if the secretions of Kp-R inhibit the growth of Kp-S and affect entry into A549 cells. To answer the question, we supplemented Kp-S suspensions with sterilized supernatants of Kp-R and calculated the ratio of entry of Kp-S. It was found that Kp-R’s supernatants did promote Kp-S’s entering the cells by 9.68 ± 0.04% (100 μL supernatants added) and by 10.04 ± 0.05% (200 μL supernatants added) (Figure 2b), and there was no significant difference between the two experimental groups. 

Whether the initial MOI was 0.1 or 1, 24 h after infection, the number of intracellular viable bacteria cells in the two strains was the same, at about 6.6–6.8 log10 CFU. Comparisons of the actual number of bacterial cells that had entered at 3 h showed that the Kp-S bacteria had a faster intracellular proliferation ability (Figure 2c). 

We used the CCK-8 assay to evaluate cell viability 3 h after infection and supernatant treatment. As shown in Figure 2d, after being infected under MOI 1 for 3 h, A549 cell viability was obviously destroyed. The cell viability of the Kp-R group was significantly higher than the Kp-S group, indicating that the cytotoxicity of sensitive strains was stronger than drug-resistant strains. For groups infected by Kp-S supplemented with 10, 20, 30 μL of Kp-R supernatants, the cell viability showed no significant difference from the Kp-S-infected groups, suggesting that Kp-R supernatants did not influence cell viability.

In summary, the strain Kp-R is more likely to adhere to epithelial cells’ surface and therefore enter the human lung epithelial cell line A549. However, the intracellular proliferation of Kp-S was faster. The difference in A549 cell viability between the two infection groups was significant, suggesting that the sensitive strain had stronger cytotoxicity. Based on the experimental results for cytotoxicity and intracellular proliferation, an MOI of 1 was used in subsequent infection experiments. 

### 2.4. Immunofluorescence Analysis of the Cytoskeleton (Actin Microfilament F-Actin) and Tight-Junction Protein Zo-1 of the Two K. pneumoniae Strains Infected A549 Cells

**Figure 3:**
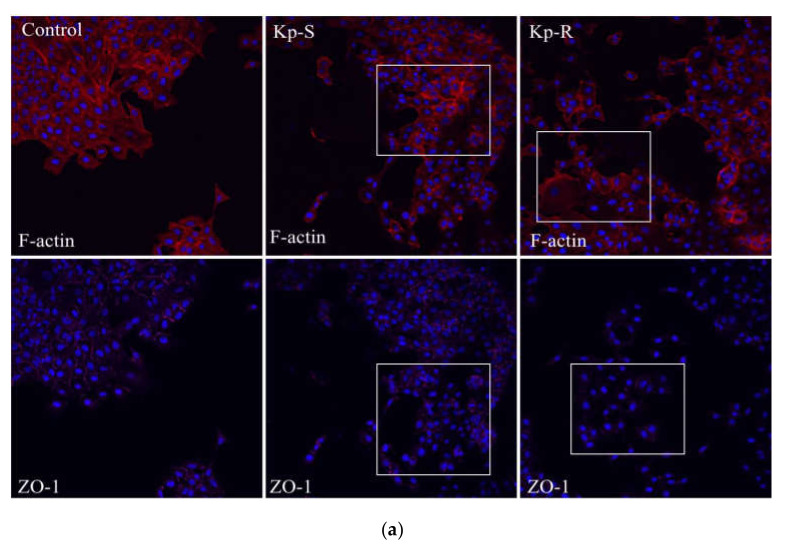

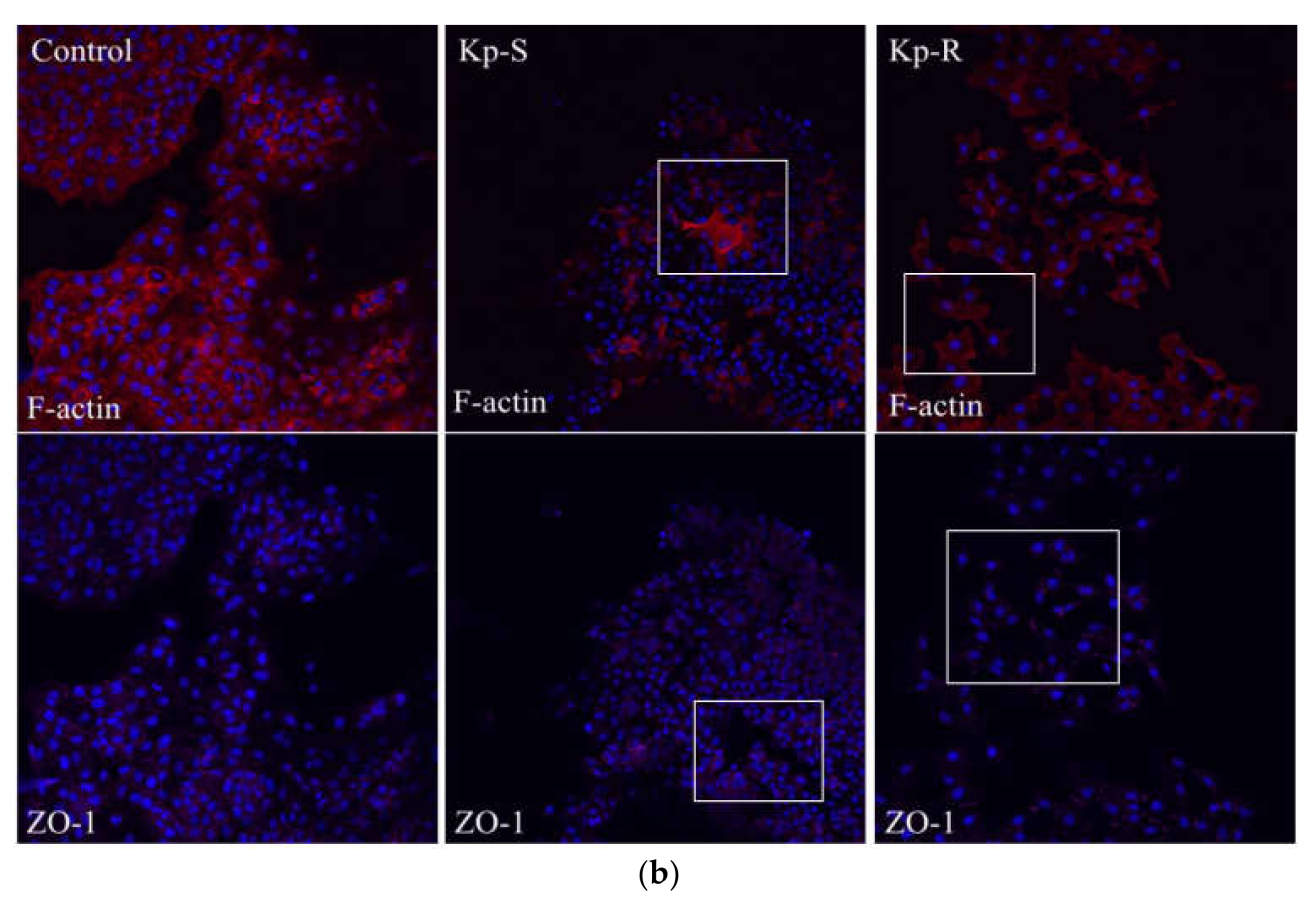
Cytoskeleton protein F-actin and tight-junction protein Zo-1 were stained in Kp-S and Kp-R-infected A549 cells. (**a**) Immunofluorescence staining of F-actin and Zo-1 12 h post-infection. (**b**) Immunofluorescence staining of F-actin and Zo-1 16 h post-infection.

The cytoskeleton can be manipulated by microbial pathogens to facilitate productive infection. Since Kp-R and Kp-S affected the viability of A549 cells, we stained the cytoskeleton F-actin and the tight-junction protein zonula occludens 1 (Zo-1). As shown in Figure 3a, the significant destruction of cytoskeleton morphology and a loss of integrity of tight junctions were observed after Kp-R and Kp-S infection for 12 h. At 16 h after Kp-R and Kp-S infection, an increased reduction in Zo-1 expression was detected and a more severe disorganized cytoskeleton was seen, as shown in Figure 3b. Comparisons between the cytoskeleton and tight junctions of the two infected groups gave the same conclusion as the CCK-8 assays, which revealed that Kp-S caused more severe damage to A549 cells than Kp-R.

### 2.5. Comparison of Metabolites Secreted by Kp-R and Kp-S

**Figure 4:**
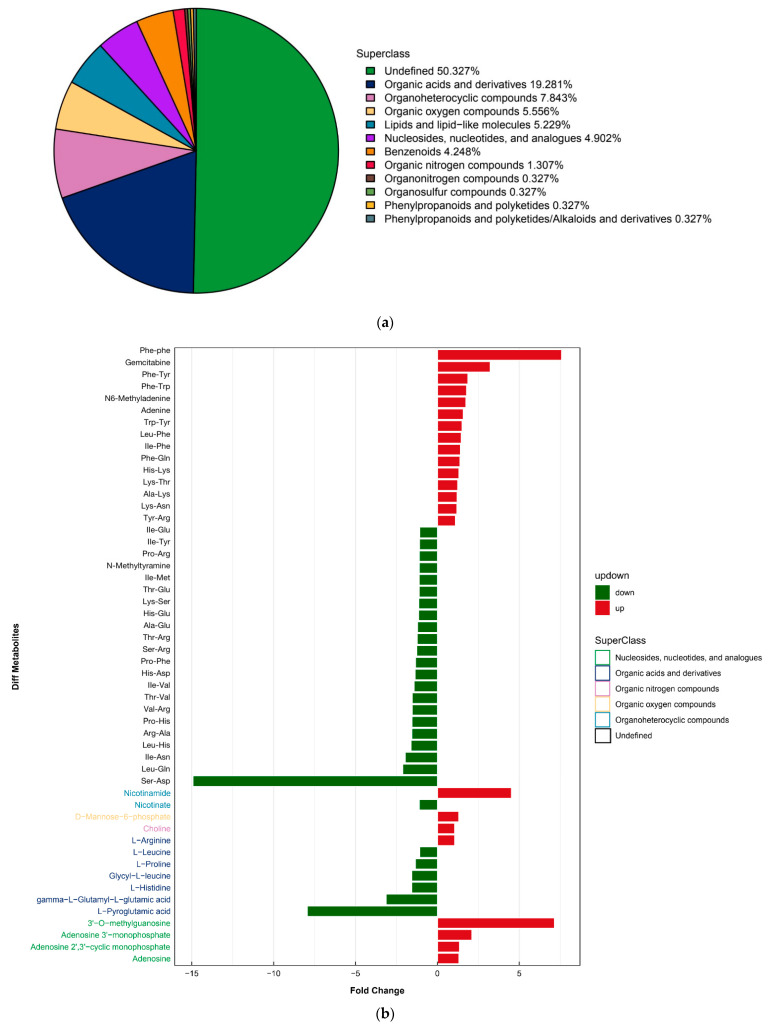

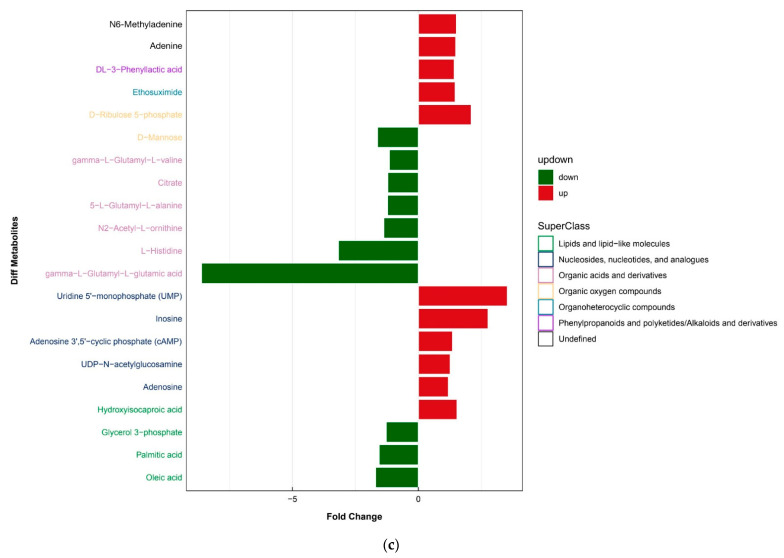
Metabolic diversity of the two *K. pneumoniae* strains. (**a**) Superclass pie chart of compounds secreted by the two *K. pneumoniae* strains. Numbers in parentheses indicate the total number of *K. pneumoniae*-associated volatile compounds for which the compound class could be determined per medium. (**b**) Analysis of significant differences in metabolite expression in positive-ion mode. (**c**) Analysis of significant differences in metabolite expression in negative-ion mode.

Unbiased metabolomic profiling of extracellular secretions showed differences of the two strains, as illustrated by a pie chart (Figure 4a). A total of 306 metabolites were identified by liquid chromatography–tandem mass spectrometry (LC–MS/MS) (*p* < 0.05) (Table S2). After normalizing to total peak intensity, the processed data were subjected to a multivariate analysis of orthogonal partial least-squares discriminant analysis (PLS-DA) for negative or positive ion labels between the two strains (Figures S2 and S3). When analyzing the differences in metabolite expression in positive-ion mode, 93 metabolites significantly increased and 94 decreased (*p* < 0.05) (Figure 4b, Table S3); whereas 89 metabolites significantly increased and 91 decreased in negative-ion mode (Figure 4c, Table S4). When KEGG enrichment analysis (Kyoto Encyclopedia of Genes and Genomes, http://www.kegg.jp/ accessed on 17 September 2020) was applied, 16 predominant molecular species were found produced by Kp-R, including inosine, adenine, adenosine, adenosine 2′, 3′-cyclic monophosphate, adenosine 3′, 5′-cyclic phosphate (cAMP), adenosine 3′-monophosphate, choline, citrate, d-mannose, d-Ribulose 5-phosphate, glycerol 3-phosphate, inosine, l-Arginine, l-Histidine, l-Leucine, l-Proline and *N*2-Acetyl-l-ornithine.

**Figure 5:**
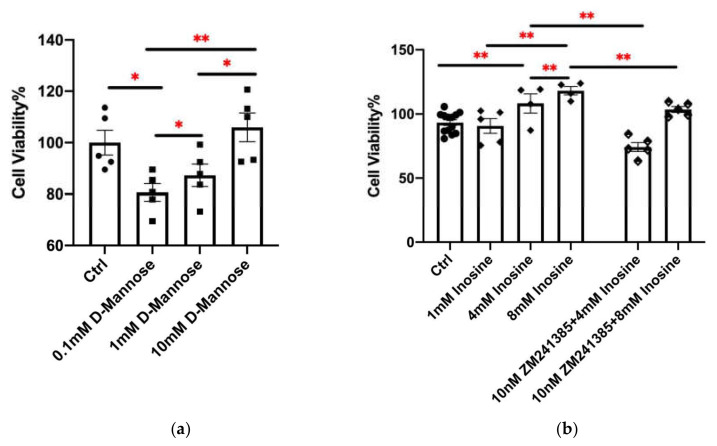
The effects of inosine and D-mannose on A549 cell viability after 3 h stimulation. (**a**) A549 cell viability was evaluated after treatment with inosine at various concentrations (1, 4, 8 mM), with or without pre-treatment with 10 nM ZM241385 for 0.5 h. (**b**) A549 cell viability was evaluated after treatment with D-mannose at various concentrations (0.1, 1, 10 mM) for 3 h. *Y*-axis represents cell viability. (*, *p* < 0.05, **, *p* < 0.01, Mean ± SE; Data analyzed by the Student’s *t*-test. The experiments were performed in triplicate).

Among these metabolites, the expression level of inosine was 2.77 times higher in resistant strain Kp-R. Inosine has proved to bind to the adenosine 2A receptor (A_2A_R). Moreover, inhibition of A_2A_R (ZM241385) can eliminate the effects of inosine [23,24,25,26]. To analyze the effect of the bacteria-secreted inosine on its virulence, we set a 1–8 mM inosine concentration gradient with or without 10 nM of the pharmacological A_2A_R antagonist ZM241385 to evaluate its effect on A549 cell viability after 3 h of stimulation (Figure 5a). It was shown that 1 mM inosine did not significantly affect cell viability, while 4 mM and 8 mM increased cell viability. When pre-treated with 10 nM ZM241385, the increase was inhibited. The results implied that inosine could be a reason for the different cell viability between Kp-R- and Kp-S-infected cells.

In addition, the secretion of D-mannose by Kp-R was 38% less than Kp-S. We stimulated cells with 0.1, 1, and 10 mM D-mannose in our experiment. As Figure 5b shows, the cell viability decreased at the concentration of 0.1 and 1 mM of D-mannose. The higher the concentration is, the slighter the decrease. When the concentration was 10 mM, the decrease disappeared. It implied that Kp-S secreted D-mannose in the concentration range that can be harmful to A549 cells while Kp-R secreted it at a higher concentration which will not affect A549 cells. In summary, both inosine and D-mannose can affect A549 cell viability, and the results were in line with the previous research [27,28,29].

## 3. Discussion

Antimicrobial resistance is an urgent threat to global health, and the development of new drugs and novel treatment strategies is highly dependent on a thorough understanding of multidrug-resistance mechanisms. Morphological changes in microorganisms under stressful conditions are the most visible parameters of bacterial adaptation [30], which may have consequences for therapeutic responses. Laar et al. compared five *K. pneumoniae* isolates using SEM but did not find any detectable morphological changes under normal culture conditions. However, stepwise-adapted *K. pneumoniae* that were resistant to cefotaxime, or polymyxin B in combination with meropenem, displayed an elongation of cells and cel- surface changes [31,32]. In this study, a thicker extracellular mucus layer was seen in Kp-R, causing the bacterial cells to adhere to each other, with the cell population forming a loose aggregate. By contrast, Kp-R cells showed a tendency toward shorter cell length and smaller BET, suggesting a reduced contact area exposed to antimicrobials, which may improve drug tolerance compared with the drug-sensitive strain.

*K. pneumoniae*, as Gram-negative bacteria, can form biofilm as a thick layer covering the bacterial surface to adapt to long-term survival and the host immune environment [12]. The glycolipids in biofilms perform many functions. Their crucial function is pathogen–host interactions, which enables bacteria to enter host cells, form agglomerates within host cells, and recruit other cells to aggregate [33]. *K. pneumoniae* has evolved the ability to attach, infiltrate and destroy host cells. Pili, capsule, endotoxin, iron carriers, antiserum immune factors, and biofilm all form the molecular basis of *K. pneumoniae* toxicity [34]. CPS is one of the main constituents of biofilms and is necessary for the virulence of *K. pneumoniae* [35]. In our study, we found that the drug-resistant strain had shown more CPS production and biofilm formation, suggesting that the bacteria survive in adverse conditions, such as in the host immune environment.

The epithelium is the lung’s first barrier against pathogens, and the adhesion and proliferation of bacteria in epithelial cells are crucial to the pathogenicity of *K. pneumoniae*. Human lung epithelial cell line A549 is a widely used cell line for studying respiratory diseases [36]. Therefore, we explored the invasive ability of drug-resistant *K. pneumoniae* with the A549 cell line. Kp-R adhered to and invaded A549 cells more effectively than Kp-S at low infection doses, which was partly due to the facilitation of CPS, as we found and concurred with previous reports [35]. However, the high cell-entry rate may not be a reason for the toxicity because the sensitive-strain-infected cells showed poorer cell viability. Compared with BET and CPS quantification, the polysaccharide mucus layer of Kp-R appeared more essential for bacterial adhesion and invasion, which was consistent with previous reports [37].

Interestingly, as the observation time extended, the intracellular proliferation ability of Kp-S was better than that of Kp-R, which implied that the damage to epithelial cells exerted by sensitive *K. pneumoniae* was related to the number of bacteria with infection progress. Cano et al. demonstrated that the cytotoxicity of epithelial cells is not directly related to bacterial adherence to host cells [38]. A large number of reports have clarified that *K. pneumoniae* produces several virulence factors, including antiphagocytic capsular polysaccharide [38,39], LPS [40,41], siderophores [42], and adhesions, but specific cytotoxic factors for host cells have not yet been determined [43].

The subcellular mechanisms involved in the *K. pneumoniae* infectious process have remained largely elusive. Direct bacterial spread is accomplished via the recruitment of the host cell cytoskeleton. Restructuring the actin component of the cytoskeleton is critical for diverse cellular processes, including endocytosis, motility, nutrient acquisition, and mitosis [39,40,44,45]. Due to their ubiquity in cells, bacterial pathogens have devised strategies to control microfilaments and Zo-1 for their benefit. To examine the subcellular alterations that occur during *K. pneumoniae* infection, we observed the microfilament protein F-actin and Zo-1 by immunostaining. F-actin is a cytoskeletal protein used as a marker to detect the cell morphology or stages of the cell cycle. Zo-1 forms tight junctions between epithelial cells and attaches to actin filaments of the cytoskeleton, which play essential an role in diverse cell behaviors including epithelial polarization, cell–cell signaling, and cell death [46]. When bacteria initiate lung infection, they also initiate an invasion of other parts of the body by disrupting tight junctions and destroying the barrier integrity. Our staining results revealed that *K. pneumoniae* induces cytoskeleton destructions and tight-junction loss in host cells, and the impacts aggravated with time. A more dramatic reduction in Zo-1 and structural failure in the cytoskeleton was observed in the infection elicited by the drug-sensitive strain, confirming the greater deterioration of structural integrity caused by the drug-sensitive strain. Our results were consistent with the previous research that validated the high-virulence strains related to drug sensitivity [44].

Pathogenic bacteria interact with immune cells in various ways. Bacterial secretions include a variety of proteins, nucleic acids, and glycolipid substances. The great potential of small molecular metabolites produced during the intracellular growth in bacteria in the transmission of immune signals is worthy of investigation. 

Despite the recent focus on *K. pneumoniae* genomics [2,45,47,48,49,50] and the epidemiology of *K. pneumoniae* infections [27,28], little attention has been paid to the pathogen metabolism, which interacts with other microorganisms or hosts in various ways. To provide a new perspective to understand bacterial pathogenicity and drug resistance, the secreted metabolites of a drug-resistant and a drug-sensitive *K. pneumoniae* strain were analyzed. Furthermore, the effects of differentially expressed metabolites on epithelial cell viability were investigated. For the first time, the way in which the secretory metabolites of *Klebsiella pneumoniae* drug-resistant strains affect host cells was analyzed, with inosine being particularly significantly upregulated and D-mannose being downregulated.

Inosine, a primary metabolite of adenosine, is an important intracellular purine nucleoside. By acting as the functional agonist of adenosine receptors, inosine is involved in regulating many physiological and pathophysiological processes. Exciting research results showed that the metabolite inosine that is produced by intestinal *B. pseudolongum* could enhance the efficacy of checkpoint blockade immunotherapy (ICB). The increased systemic translocation of inosine resulting from impacted gut-barrier function due to ICB induced anti-tumor immunity in T cells and could also directly affect tumor cells [24]. Therefore, we treated A549 cells with inosine and confirmed that cell viability was affected. Then, we treated the cells with an antagonist of A_2A_R (ZM241385) to eliminate inosine’s effects. As we found, after the inhibition of A_2A_R, cell viability decreased, which implied the inosine–A_2A_R signaling pathway could also modulate epithelial cells. However, further investigation is needed.

D-mannose as a natural sugar was confirmed to act as an ‘antibiotic-like’ option in the prevention and treatment of uropathogenic *Escherichia coli*. As our experiment confirmed, D-mannose decreased A549 cell viability at the concentration of 0.1 and 1 mM, but the influence no longer existed when the concentration was 10 mM. The results suggested that Kp-S secreted D-mannose at a specific level related to cytotoxicity.

Interestingly, we conducted a global study on the secretory metabolome of the drug-resistant strain and found that inosine was secreted at a high level while that of D-mannose was low. Furthermore, A549 cells showed a consistent reaction to drug-resistant bacteria, the supernatant of the drug-resistant strain, inosine, and D-mannose.

## 4. Materials and Methods

### 4.1. Bacterial Strains and Culture Conditions

In this study, a drug-sensitive *K. pneumoniae* strain ATCC 27736 and an ESBL strain of *K. pneumoniae* ATCC 700603 (Manassas, VA, USA) were used. The *K. pneumoniae* strains were grown in LB medium with shaking (220 rpm) at 37 °C [51]. For long-term storage, they were kept in 30% glycerol solutions at −80 °C.

### 4.2. Bacterial Growth Kinetics In Vitro

The bacterial growth curve was drawn over 30 h by measuring the OD 600 at 3 h intervals. For growth kinetics analysis, two strains grew to OD 600 of 0.2. A total of 500 μL of the preculture was inoculated into 50 mL of LB medium and cultured on an orbital shaker (220 rpm, 37 °C). The OD 600 was recorded at the indicated time points for 30 h. For competitive growth analysis in vitro, two strains grew to the logarithmic growth phase at 600 nm (OD 600) of 0.8–1.0, then 200 μL cultures of each strain were mixed and inoculated in 4 mL LB medium at 37 °C. After 12 h of mixed culture, the CFU of Kp-R was enumerated by spot-plating serial dilutions on LB agar plates adding 50 μg/mL kanamycin, and the CFU of Kp-S was calculated by subtracting the CFU enumerated on kanamycin supplemented LB agar plates from CFU of the antibiotic-free LB agar plates. All experiments were repeated three times.

### 4.3. Scanning Electron Microscopy Analysis

The selected Kp-R and Kp-S cultures (5 mL each) grew to the mid-log phase with an OD 600 of 0.8. The bacterial cells from each culture were washed with potassium-phosphate buffer (50 mM, pH 7.0), and two droplets of heparin sodium were added. Bacterial cells were then fixed by immersion in 3% glutaraldehyde in potassium-phosphate buffer overnight at 4 °C. Following two washes with buffer, the specimens were dehydrated with ethanol (*v/v*) ranging from 10% to 90% and stored in 100% ethanol. For SEM, the specimens were dried to a critical point, coated with gold, and observed under a scanning electron microscope (SU5000; Hitachi, Tokyo, Japan). Cellular widths and lengths were measured with Image J. BET was calculated by the formula (2πr^2^ + 2πrh)/πr^2^h [52].

### 4.4. CPS Quantification of K. pneumoniae

CPS, which mainly contains uronic acid, was quantified by a phenol-extraction method, as previously described [53]. The strains grew in 5 mL LB medium on an orbital shaker (220 rpm, 37 °C) overnight and were harvested by centrifugation at 4000 rpm for 10 min. The cell pellets were washed 3 times with distilled water and resuspended in 500 μL distilled water. The number of strains was calculated as mentioned above. A total of 1.2 mL of 12.5 mM tetraborate (Solarbio, Beijing, China) in concentrated sulfuric acid was added to 100 μL of the cell suspensions. After being vigorously vortexed, the samples were boiled for 5 min. Once they cooled to room temperature, 20 μL of 0.15% 3-hydroxydiphenyl (Sigma-Aldrich, St. Louis, MO, USA) was added. Then, the absorbance at 570 nm was measured.

### 4.5. Biofilm-Formation Assay

Strains were statically cultured in 96-well cell plates (Nest, Wuxi, Jiangsu, China) to measure biofilms. After culturing the strains overnight, the LB medium was removed, and the bacterial cells were gently washed with 200 μL distilled water. The plates were air-dried for 40 min. Biofilms were stained with 100 μL 0.1% crystal violet diluted in distilled water for 30 min. Then, the wells were gently washed 3 times with distilled water. The image was taken for visual observation and for quantitation, OD 570 was detected through a microplate reader (BioTek Gen5; BioTek Instruments, Winooski, VT, USA) [54].

### 4.6. Antimicrobial Susceptibility Testing

The antimicrobial susceptibility was experimentally determined using a commercial bacterial identification system (MicroScan WalkAway system, Dade MicroScan, Inc., West Sacramento, CA, USA) with Neg/BP/Combo 30-B1017-306E combination panels following the Clinical and Laboratory Standards Institute (CLSI) guidelines [55,56]. *E. coli* ATCC 25922 and *Pseudomonas aeruginosa* ATCC 27853 were used as quality-control strains. The susceptibility of the *K. pneumoniae* strains to antibiotics (listed in Table S1) was determined according to the recommendations of the CLSI. The confirmatory test was performed using the double-disk-diffusion (DD) testing method. Mueller–Hinton agar plates (MHA, Thermo Fisher, Waltham, MA, USA) were inoculated with the two *K. pneumoniae* strains. Ceftazidime and ceftazidime–clavulanate disks were placed 30 mm apart on MHA and incubated at 35 °C for 16 h. Enhancement of the inhibition zone in the area between the discs indicated the ESBL test result. The experiments were repeated 2 times.

### 4.7. Selection of Secreted Compounds

The supernatants of the two strains were collected after they had grown to mid-log phase with an OD 600 of 0.8 (the approximate CFU was 5.7 × 10^8^) and analyses were performed using an UHPLC (1290 Infinity LC, Agilent Technologies, Santa Clara, CA, USA) coupled to a quadrupole time-of-flight (AB Sciex Triple TOF 6600) in Shanghai Applied Protein Technology Co., Ltd. (Shanghai, China). After sample preparation and normalization to total peak intensity, the processed data were analyzed using the R package (ropls), including Pareto-scaled principal component analysis (PCA) and orthogonal partial least-squares discriminant analysis (OPLS-DA). Seven-fold cross-validation and response-permutation testing were used to evaluate the robustness of the model. The variable importance in the projection (VIP) value of each variable in the OPLS-DA model was calculated to indicate its contribution to the classification. Metabolites with a VIP value >1 were further applied to the Student’s *t*-test at the univariate level to measure the significance of each metabolite, and a *p* value less than 0.05 was considered statistically significant.

### 4.8. Strain Supernatants Collection

Kp-R and Kp-S were cultured to OD 600 = 0.6 (at this absorption value, the approximate CFU is 5 × 10^7^), and the supernatants were collected by centrifugation. Aliquots were equally divided into 200 μL and stored in an −80 °C refrigerator.

### 4.9. Cell Culture and K. pneumoniae Infection

Human lung epithelial cell line A549 from the American Type Culture Collection (ATCC CCL-185) was incubated in Dulbecco’s modified Eagle’s medium (DMEM, Solarbio, Beijing, China) supplemented with 10% fetal bovine serum (FBS; Thermo Fisher, Waltham, MA, USA) in 5% CO_2_ incubator.

A549 cells were seeded in 24-well plates at a density of 1 × 10^5^ cells per well. After 24 h, cell-culture medium containing Kp-R and Kp-S at MOI of 0.1, 1 was added to the wells with or without 100 μL of Kp-R supernatants. After 3 h of inoculation, the medium was removed, and the cells were washed 3 times with sterile phosphate-buffered saline (PBS) and lysed with 1 mL 0.1% Triton X-100 for 15 min. Initial CFU and CFU (3 h) of Kp-R and Kp-S was enumerated by spot-plating serial dilutions with the lysates spread on LB agar plates (Solarbio, Beijing, China). At 16 h and 24 h post-infection (POI), A549 cells were lysed with 1 mL 0.1% Triton X-100, and CFU (16 h or 24 h POI) was enumerated.

### 4.10. Metabolites and Strain Supernatants Stimulation

A549 cells were seeded in 96-well plates at 5000 cells per well. After 24 h, the A549 cells were separately treated with 1–8 mM inosine (Abmole Bioscience Inc., Houston, TX, USA) diluted in PBS. Inosine was inhibited by a 30 min pre-treatment of the A_2A_R antagonist ZM241385 (Abmole Bioscience Inc., Houston, TX, USA) at a concentration of 10 nM. D-Mannose (Solarbio, Beijing, China) was diluted in the cell medium at a concentration of 0.1–10 mM. For strain supernatant stimulation, A549 cells were treated by addition of 10, 20, 30 μL of supernatants to the medium, with or without the Kp-S (MOI 1).

### 4.11. Cell Viability Assay

A549 cells seeded in 96-well plates were incubated with the *K. pneumoniae* strains, supernatants, inosine (Abmole Bioscience Inc., Houston, TX, USA), or D-mannose (Solarbio, Beijing, China). Then, CCK-8 was used to detect the cell viability at OD 450 with a microplate reader (BioTek Gen5; BioTek Instruments, Winooski, VT, USA), according to the instructions of Cell Counting kit-8 (M4839, 500 test/5 mL, Abmole Bioscience Inc., Houston, TX, USA).

### 4.12. Immunofluorescence Assay

After fixation in 4% paraformaldehyde, permeabilization in 0.1% Triton X-100 and blockade with 1% bull serum albumin, the cells were incubated with anti-Zo-1 (Beyotime, Beijing, China) at 4 °C overnight and then goat anti-rabbit IgG secondary antibody (Abcam, Cambridge, UK) and Actin-Tracker-Green-488 (Beyotime, Beijing, China) for 1 h at room temperature. After removing the secondary antibodies, DAPI (Beyotime, Beijing, China) was applied to the cells. Images were taken with a fluorescence microscope (Olympus, Hamburg, Germany).

### 4.13. Statistical Analysis

Statistical differences were analyzed by Student’s *t*-test between every two groups and one-way analysis of variance (ANOVA) for multiple groups. The values where *p* < 0.05 were taken as being statistically significant. Statistical data analysis was performed using GraphPad Prism software (version 9.0.2, La Jolla, CA, USA).

## 5. Conclusions

In conclusion, we compared the physiological, biochemical, and cultural characteristics of a drug-resistant *K. pneumoniae* strain (Kp-R) with a drug-sensitive strain (Kp-S). A thicker extracellular mucus layer, more biofilm, and CPS production appeared in Kp-R. When the two strains were co-cultured, Kp-R had an advantage in growth competition. We also attempted to analyze extracellular metabolites of the two strains by LC-MS/MS, and 16 differentially expressed metabolites were identified, including inosine, adenine, adenosine, adenosine 2′, 3′-cyclic monophosphate, adenosine 3′, 5′-cyclic phosphate (cAMP), adenosine 3′-monophosphate, choline, citrate, D-mannose, D-Ribulose 5-phosphate, glycerol 3-phosphate, inosine, L-Arginine, L-Histidine, L-Leucine, L-Proline and N2-Acetyl-L-ornithine. Moreover, we infected the A549 cell line with Kp-R and Kp-S to investigate the pathogen–host interaction in *K. pneumoniae*-associated pneumonia. Kp-R showed a higher entry ratio into A549 cells than Kp-S. However, at 16 h and 24 h POI, Kp-S proliferated faster than Kp-R. Kp-R supernatants were found to be helpful to bacterial entry. A549 cell viability was measured after Kp-R and Kp-S infection, and the Kp-S-infected group showed poorer cell viability, which is in accordance with the immunostaining results of Zo-1 and F-actin. The differentially secreted metabolites inosine and D-mannose affected A549 cell viability in a specific concentration range. Specifically, inosine increased the cell viability, which its antagonist ZM241385 could eliminate, and D-mannose decreased the cell viability.

## Data Availability

The data presented in this study are all available in the main text and Supplementary Materials.

## Supplementary Materials

The following supporting information can be downloaded at: https://www.mdpi.com/article/10.3390/molecules27092994/s1, Figure S1: Growth curve in BHI medium. Table S1: Antimicrobial susceptibility of the two *K. pneumoniae* strains. Figure S2. PCA analysis of the positive ion model for the two samples. Figure S3. PCA analysis of the negative ion model for the population samples. Table S2: 306 metabolites identified by LC-MS/MS in total. Table S3: Differentiated metabolite selection in positive ion mode. Table S4: Differentiated metabolite expression in negative ion mode.

## Sample Availability

Samples of the compounds inosine and D-mannose are available from the authors.
