# Supplementary material for: Inosine and D-Mannose Secreted by Drug-Resistant Klebsiella pneumoniae Affect Viability of Lung Epithelial Cells
Source: Molecules. 2022 May 6;27(9):2994. doi: 10.3390/molecules27092994 (PMC9106066; doi:10.3390/molecules27092994)
Supplement: Supplementary file 1 [file molecules-27-02994-s001.zip › molecules-1681005-supplementary.pdf]

Supplementary Materials

Inosine and D-Mannose Secreted by Drug-Resistant *Klebsiella pneumoniae* Affect Viability of Lung Epithelial Cells

Yuhan Zhang [1,2], Ziwei Zhou [3], Wenxuan Xiao [3], Yuting Tang [3], Wei Guan [1,2], Jiang Wang [1,2], Farui Shu [3], Jiaqi Shen [3], Shaoyan Gu [1,2], Lu Zhang [3], Qingzhong Wang [4,*] and Lixin Xie [1,2,*]

[1] College of Pulmonary and Critical Care Medicine, Chinese People's Liberation Army General Hospital, Beijing 100853, China; zhangyuhannku@foxmail.com (Y.Z.); guanwei91@126.com (W.G.); albert_19891117@163.com (J.W.); shaoyan.gu@outlook.com (S.G.)
[2] Medical School of Chinese People's Liberation Army, Beijing 100853, China
[3] State Key Laboratory of Genetic Engineering, Institute of Genetics, School of Life Science, Fudan University, Shanghai 200437, China; zhou_ideal@163.com (Z.Z.); xiao-xwx@foxmail.com (W.X.); 18321656189@163.com (Y.T.); 16307110154@fudan.edu.cn (F.S.); 18307110040@fudan.edu.cn (J.S.); zhanglu407@fudan.edu.cn (L.Z.)
[4] Shanghai Centre for Clinical Laboratory, Shanghai 200126, China
* Correspondence: email: wangqingzhong@sccl.org.cn (Q.W.); xielx301@126.com (L.X.)

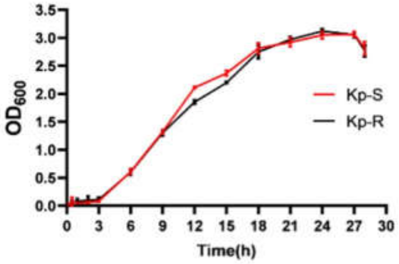

Figure S1. Growth curve in BHI medium.

Table S1. Antimicrobial susceptibility of the two *K. pneumoniae* strains.

Antimicrobial agent	MIC (mg/ml) for *K. pneumoniae* 27736	Susceptibility S/I/R*	MIC (mg/ml) for *K. pneumoniae* 700603	Susceptibility S/I/R*
Ampicillin/Sulbactam	<8/4	S	>16/8	R
Amoxicillin/K.Clavulanate	<16	S	32	R
Ampicillin	16	I	>16	R
Aztreonam	<4	S	>16	R
Ceftriaxone	<1	S	>32	R
Ceftazidime	<1	S	>16	R
Ceftazidime/Ceftriaxone	<0.25	S	>2	R
Cefaclor	<2	S	>32	R
Cefaclor/Ceftriaxone	<0.5	S	>4	R
Cefamendole	<8	S	>16	R

Cefazolin	<8	S	>16	R
Ciprofloxacin	<1	S	>2	R
Cefepime	<4	S	>16	R
Cefuroxime	<4	S	>16	R
Ertapenem	<1	S	>4	R
Nitrofurantoin	<32	S	>64	R
Gms.Screen	<4	S	<4	R
Imipenem	<1	S	8	I
levofloxacin	<2	S	>4	R
Meropenem	<1	S	>8	R
Penicillin/Tazobactam	<16	S	>64	R
Trimethoprim/Sulfa	<2/38	S	>2/38	R
Ticarcillin/Clavulanic acid	<2	S	<2	S
Tobramycin	<4	S	>8	R

*S, susceptible; I, intermediate; R, resistant.

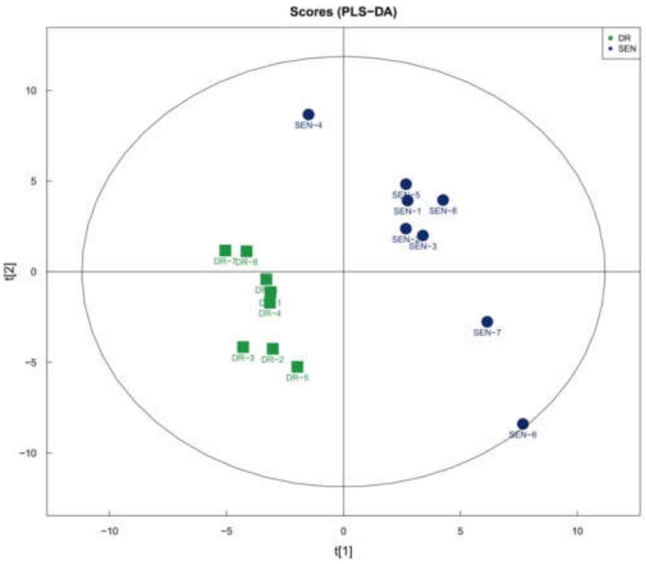

Figure S2. PCA analysis of the positive ion model for the two samples.

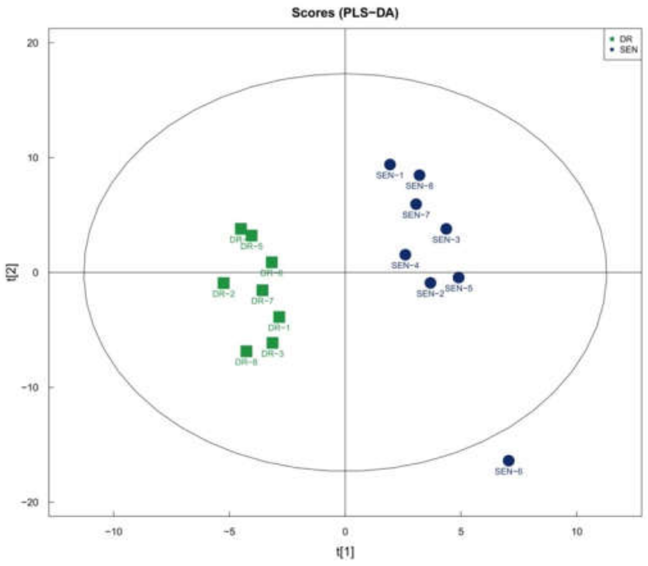

Figure S3. PCA analysis of the negative ion model for the population samples.

Table S2. 306 metabolites identified by LC-MS/MS in total.

Class	NO. of identified metabolites
Undefined	154
Carboxylic acids and derivatives	50
Organooxygen compounds	17
Benzene and substituted derivatives	12
Fatty Acyls	12
Indoles and derivatives	8
Pyridines and derivatives	6
Pyrimidine nucleotides	6
Hydroxy acids and derivatives	5
Diazines	4
Organonitrogen compounds	4
Imidazopyrimidines	3
Purine nucleosides	3
Purine nucleotides	3
Steroids and steroid derivatives	2
5'-deoxyribonucleosides	1
Amines	1
Carboximidic acids and derivatives	1
Glycerophospholipids	1

Keto acids and derivatives	1
Nucleoside and nucleotide analogues	1
Organic sulfonic acids and derivatives	1
Organic sulfuric acids and derivatives	1
Phenols	1
Phenylpropanoic acids	1
Phenylpropanoic acids/Tropane alkaloids	1
Prenol lipids	1
Pteridines and derivatives	1
Pyrrolidines	1
Quinolines and derivatives	1
Ribonucleoside 3'-phosphates	1
Thioethers	1
Total number	306

Table S3. Differentiated metabolite selection in positive ion mode.

ID	adduct	Name	VIP	Fold change	p-value	m/z	rt(s)
M251T130			5.037859257	25.07166674	0.336416793	251.10273	129.844
M249T419			0.197759827	19.27217759	0.326301433	249.05066	418.8025
M446T367			2.435102739	13.29456097	0.338758202	446.14904	366.7445
M325T422			4.251670249	12.19147098	1.01593E-16	325.04323	422.136
M376T32			3.578152828	10.01965173	0.02341697	376.25948	32.206
M618T32			3.387404472	8.599943111	0.02318054	617.53578	32.206
M352T262			1.120307518	8.419488431	2.95089E-06	352.01886	262.064
M213T422			3.067247086	8.303166878	4.36548E-16	213.01553	422.447
M393T32			3.071647512	8.266401546	0.025268076	393.28572	32.4145
M314T263			2.377913356	8.212738999	8.56879E-07	314.0636	262.811
M313T125	(M+H)+	Phe-Phe	3.998573199	7.585636308	1.39718E-12	313.15457	125.385
M364T449	(M-2H+3Na)+	3'-O-methylguanosine	5.455682086	7.141164852	2.65703E-15	364.06547	448.724
M215T278			0.661925419	7.092778912	0.161013462	215.0936	277.788
M562T270			1.112781105	6.858221511	0.347889622	562.34352	269.57
M152T449			6.971844202	6.632286437	1.33238E-14	152.05633	448.724
M261T237_1			2.9167672	6.607917626	0.166337323	261.1444	236.829
M386T449			2.031383911	6.367919884	3.31991E-14	386.04716	448.533

M551T276			1.50209763	6.308158453	0.145611416	551.33563	276.494
M230T354			1.156987284	6.010635794	0.330758772	230.07731	354.273
M175T375			1.235320583	5.083769732	0.331506812	175.08966	374.944
M356T404_2			1.468561511	4.886821611	0.301180208	356.10648	404.245
M612T411			0.768818702	4.666583168	0.325184105	612.12471	411.0995
M739T447			0.735826232	4.62179909	3.06878E-10	739.20454	447.0165
M123T60	(M+H)+	Nicotinamide	20.40138827	4.520378492	3.47106E-05	123.05498	60.053
M380T267			1.400619365	4.064941493	1.3887E-08	380.1217	266.773
M136T187			1.409339367	3.957918412	0.278084063	136.07468	186.841
M155T75			2.683516719	3.785021094	0.290583408	155.11738	74.799
M205T277			1.373106774	3.779435059	0.351612804	205.11783	276.76
M360T396			2.354972268	3.750872561	6.24543E-08	360.1497	395.842
M337T291			0.31262251	3.692019846	0.142407502	337.15015	291.321
M423T493			2.314852821	3.616306126	4.01896E-11	423.08979	493.092
M231T428			1.120398083	3.608140391	0.37103559	231.13429	428.365
M467T252			0.743638833	3.504003598	0.325637014	467.15057	251.72
M325T396			1.757656655	3.494308731	4.4687E-09	325.11305	395.647
M244T60			2.600470991	3.485824534	0.030306206	244.26353	59.747
M162T287			0.897628063	3.324778866	2.39924E-08	162.07551	287.151
M1020T450			0.975503321	3.300155015	9.02043E-09	1020.33322	449.978
M288T40			14.34739053	3.256178063	0.058693028	288.29021	39.608
M549T405	(2M+Na)+	Gemcitabine	1.73408197	3.221846662	2.09042E-12	549.13383	404.924
M186T100			0.700023494	3.137604747	0.320746417	186.05301	100.3625
M445T493			0.702408798	3.13683437	1.29133E-09	445.07138	492.863
M344T392			0.692608986	3.126113715	0.351482952	344.08864	392.218
M231T240			0.886839554	3.111549229	0.337304798	231.0767	239.7645
M203T162			0.748270515	3.069879185	0.16122254	203.13956	161.6915
M409T297			1.179716412	3.045758426	0.346709848	409.14801	296.511
M225T42_2			3.567301577	3.0296437	0.160902227	225.12307	41.835
M444T441			0.828090439	2.998514588	0.09579666	444.15931	440.658
M235T241			5.460247572	2.991579974	0.250720745	235.11869	241.202
M222T163			0.826228851	2.931924724	0.354665055	222.14874	162.5175
M307T423			0.786245427	2.892326002	1.00914E-07	307.03263	422.74
M501T432			0.38754689	2.880366544	0.141290986	501.16136	432.1965
M389T285			0.364302646	2.842438297	0.044512043	389.14962	285.096
M235T43			1.180535733	2.7098593	0.311071926	235.14341	43.145
M386T445_1			0.250332597	2.684367376	0.342130854	385.98553	444.6415
M386T477			1.177916937	2.672977677	2.02212E-12	386.0149	477.052
M571T405			0.761089441	2.664175928	6.51982E-10	571.11526	404.96
M322T262			1.910206931	2.662060011	2.03128E-05	322.05442	262.042
M481T238			0.414509341	2.639431123	0.283714932	480.80984	238.2625
M113T160			1.494014379	2.580364232	8.33434E-06	113.03371	159.999
M213T182			3.578360723	2.485632556	0.000370659	213.15954	182.2785
M163T397_2			0.931220895	2.446305426	2.49883E-08	163.05956	396.518
M722T316_2			0.781966917	2.417915258	0.331777531	722.1376	315.9075
M80T61			4.116740912	2.372850352	0.00036209	80.04908	60.774
M496T191_2	(M+H)+	1-Palmitoyl-sn-glycero-3-phosphocholine	0.748953498	2.356265872	1.54866E-05	496.3384	190.544

M468T195	(M+H)+	1-Myristoyl-sn-glycero-3-phosphocholine	0.830723369	2.336153313	0.000141039	468.30782	194.588
M431T125			0.518769038	2.333783942	0.343923989	431.15623	124.592
M138T45	(M+H)+	4-Aminobenzoate	1.532575711	2.331894315	0.172358146	138.05436	45.241
M177T269			1.542525952	2.2919033	0.122542534	177.13421	268.861
M434T480			0.603386718	2.282018755	0.141811922	434.15153	480.272
M239T224			0.300794076	2.262883143	0.356085931	239.08475	223.81
M461T144			1.255308263	2.252208743	1.17675E-05	461.17638	143.6
M185T49			2.405969259	2.250481698	0.005996241	185.09218	49.4
M691T437			0.369865928	2.240135818	0.317506381	691.13888	437.2915
M316T39			4.950799456	2.193843932	0.142221966	316.32079	38.528
M594T284			0.820382344	2.18185378	0.107997461	594.28055	284.284
M407T263			1.058989342	2.180521292	0.001596244	407.19625	263.474
M389T487_2			0.470499482	2.11614084	0.28648909	389.17331	487.045
M432T429			0.37074847	2.113682816	0.287954749	432.15497	428.749
M348T413	(M+H)+	Adenosine 3'-monophosphate	7.024588958	2.1042837	9.38841E-13	348.07105	413.294
M365T392			3.969166482	2.094085766	4.12731E-12	365.10509	392.135
M472T331			0.518898467	2.087526383	6.88169E-05	472.0214	331.0175
M255T312			0.383336101	2.08172544	0.365491347	255.22614	311.7955
M81T121			4.11061247	2.08069947	1.6244E-05	81.04415	121.348
M86T197			0.447265647	2.078431589	0.322710379	86.05958	196.997
M656T327			0.828796789	2.067693822	0.219242352	655.70137	326.513
M451T136			1.121004632	2.065706953	0.003056826	451.20862	135.509
M113T123			2.205200639	2.060784679	0.000393893	113.07037	123.458
M261T111			5.40710055	2.052995856	0.071528122	261.12564	111.333
M370T413			2.03187725	2.042755731	4.02231E-10	370.05221	412.758
M352T157			1.837784633	2.04064109	5.63645E-05	352.16522	156.923
M390T331			1.295663919	2.026752158	2.8974E-09	390.01808	331.265
M314T480			0.499786751	2.012349376	0.353871702	314.07424	479.983
M307T296			2.321782944	2.008579431	1.18729E-08	307.0323	295.996
M243T39_1			0.558065741	0.50000102	0.13369484	243.05596	38.8055
M256T367			2.018747294	0.499380374	1.72764E-06	256.08869	366.629
M116T24			1.863310781	0.495458195	0.025882274	116.05214	24.259
M325T356			0.823549954	0.494846696	9.37163E-11	325.0771	355.7
M152T229_1			0.71634978	0.493685013	0.000220282	151.59047	229.22
M332T258			0.591714508	0.491545925	0.002636378	332.10022	258.4795
M212T185			0.882658561	0.487270221	0.17601374	212.09168	184.8485
M502T395			0.655174066	0.481267753	0.175907389	502.18758	395.025
M185T124			2.632191588	0.480572989	0.160949471	185.12791	124.451
M570T37			1.0695945	0.480346326	0.037517858	570.45719	37.275
M227T362			1.855991384	0.479395183	0.000242808	227.11373	361.9475
M526T36			1.091116242	0.479186809	0.060142607	526.43102	36.058
M314T372			1.152399858	0.479111594	0.361511673	314.14609	371.723
M734T642			2.472538741	0.478258483	0.062354941	733.54161	641.885
M249T474			1.783626875	0.476889401	8.61149E-13	249.07156	473.6445
M689T242			1.569399343	0.475256471	7.45435E-05	689.38993	241.564
M261T411			0.575251295	0.472426622	0.335224494	261.07231	411.2225
M277T391			0.58454065	0.470710988	0.000204647	277.07725	391.485

M260T291	(M+H)+	Leu-Gln	9.054064239	0.470666498	6.05458E-14	260.16066	291.375
M110T446			8.401803531	0.469859033	0.001175839	110.07106	446.1705
M560T391			0.637199619	0.468823588	0.378267005	560.34046	390.859
M482T35			0.971388797	0.466800128	0.075110511	482.4047	34.7785
M382T228_2			0.760801011	0.46302644	7.43485E-08	382.21965	228.344
M185T427			0.673008873	0.461747329	3.9465E-06	185.05525	427.343
M468T35			0.84016138	0.461282566	0.038280344	468.38869	34.746
M397T288			1.038063141	0.460749084	0.338215669	397.24353	288.37
M258T427			1.511428419	0.460358676	5.63522E-06	258.10912	426.705
M188T300			0.690198399	0.460267593	0.276790884	188.1108	299.651
M241T358			0.819608693	0.459245736	1.90815E-05	241.02963	357.763
M235T73			1.260755932	0.45703056	0.372873021	235.07455	72.876
M567T351_1			0.445279366	0.454396251	0.181991115	566.6756	351.154
M125T46			1.025726661	0.447340374	0.359199811	125.10667	45.6005
M250T436_2			1.425009569	0.445597269	0.000858102	250.09232	436.04
M292T349			1.303147753	0.440728942	9.84491E-11	292.08778	349.441
M262T427			4.869478448	0.440022648	9.80069E-07	262.10376	427.4305
M804T316_2			0.678001304	0.439894393	0.148428439	804.14174	316.0995
M262T459			1.43215561	0.43801852	0.3414703	262.10424	458.9
M345T302			0.279110501	0.435868118	0.171883718	345.02819	301.73
M544T370			0.897959986	0.434551824	0.347824369	544.34539	370.087
M415T35			7.846558647	0.434254547	0.025185628	415.21148	34.644
M771T392_2			0.462761802	0.434133607	0.379401586	771.43222	392.221
M291T373			1.443705578	0.433676553	4.05571E-07	291.09217	373.338
M240T357			1.263197396	0.431227083	0.000132612	240.09547	357.457
M313T304			1.097834785	0.428408754	2.81703E-05	313.12448	303.865
M202T282_2			1.044843597	0.425793013	0.089509996	202.0495	282.203
M340T291_1			1.176859867	0.423206344	0.362600037	340.15021	290.635
M772T385_2			0.52484268	0.414812351	0.338915308	772.45401	385.368
M613T243			3.848508025	0.408487495	5.48381E-05	613.3914	242.615
M556T37			1.019414471	0.402248987	0.00947843	556.44041	37.3115
M506T25			0.947565418	0.400984937	0.336368739	506.2552	24.87
M252T311			1.054126792	0.397487696	0.004029244	252.16803	311.048
M563T313_1			0.826844212	0.394214618	0.150816728	562.75994	312.724
M485T449			4.491002503	0.390847165	0.042126986	485.3078	448.9395
M270T349			1.63189729	0.386927042	7.5178E-08	270.10639	349.422
M361T398			2.046530191	0.373524159	0.329623306	361.24435	398.119
M263T470			5.574628849	0.373277345	9.68625E-13	263.08768	469.586
M521T474			0.837883329	0.367136092	0.32178049	521.15468	474.372
M180T39			0.805765727	0.364027988	0.414049257	180.12079	38.585
M216T39			0.737303306	0.361913732	0.084623202	216.06558	38.9865
M271T410			1.032770737	0.359593506	0.300708434	271.12882	410.458
M202T115			1.05201708	0.356435342	0.311249629	202.15502	114.789
M345T277			1.259897853	0.353569359	0.357793999	345.18612	276.713
M212T37			1.996958446	0.353428754	1.29383E-07	212.12742	37.328
M203T285_1			0.897705091	0.352085015	0.328074446	203.04808	284.954
M285T469			1.542441228	0.347713637	1.36604E-12	285.06947	469.31
M200T429			0.603516739	0.346290377	0.202322343	200.05527	429.158

M373T239			0.646618684	0.339607074	0.296933106	373.18713	238.8905
M384T125_1			0.207989451	0.334025834	0.128662522	384.16879	125.466
M505T434			0.685034478	0.322160209	0.182968725	505.17658	434.049
M277T467	(M+H)+	gamma-L-Glutamyl-L-glutamic acid	3.489516116	0.319211019	1.73416E-10	277.10355	467.48
M175T215			0.826470181	0.314523709	0.135679391	175.07103	215.323
M764T345			1.830501287	0.287247596	0.356888789	763.85112	345.186
M130T239			0.495455695	0.283299403	0.312306608	130.04926	239.069
M301T249_3			1.125946215	0.281525574	3.20574E-06	301.09455	248.952
M159T97			0.794785644	0.278017157	0.386280157	159.13159	97.251
M248T355			0.929002076	0.2742188	0.141057911	248.06145	354.678
M349T371			1.194306569	0.261911264	0.359258128	349.04966	370.782
M516T42			0.648994111	0.260139967	0.346328198	516.37186	41.768
M299T437			0.171300428	0.251073347	0.114255901	298.64789	436.853
M221T237			0.698243225	0.216590893	0.329386322	221.12802	236.9345
M276T426			1.12690969	0.201457836	0.167174767	276.19229	425.642
M529T251_1			0.890219261	0.178803108	0.32503457	528.79735	250.54
M439T236			0.579396578	0.171483577	0.163269977	439.25476	236.214
M489T30			1.910030808	0.155100907	0.299634128	489.22544	30.184
M283T79			1.31857845	0.152475702	0.330616094	283.21825	78.9735
M183T48			1.838698754	0.148656574	0.158871211	183.05837	48.2665
M259T467	(2M+H)+	L-Pyroglutamic acid	4.74135962	0.125929022	1.41698E-11	259.09266	466.73
M501T237			1.009208114	0.096582019	0.342127437	501.14594	236.8135
M254T98			1.767339184	0.084458124	0.134818412	254.21156	97.842
M244T125			2.833646244	0.079865735	0.150343346	244.3392	124.713
M175T405			1.745475933	0.073968791	0.344212952	175.07092	404.972
M221T428	(M+H)+	Ser-Asp	4.169586166	0.067004167	1.15875E-09	221.07674	428.1715
M198T40			2.13884214	0.063184941	0.134813354	198.05362	40.458
M251T129			0.315868692	0.054228768	0.326990084	251.06954	129.093

Table S4. Differentiated metabolite expression in negative ion mode

ID	adduct	Name	VIP	Fold change	p-value	m/z	rt(s)
M456T249			1.964145439	0.018851001	0.332543218	456.15879	249.49
M231T392			9.86439809	0.04320567	1.99327E-16	231.09832	392.136
M219T429			6.616191644	0.049782491	2.03403E-14	219.06186	428.734
M526T299_2			3.204317287	0.095952984	0.330434138	526.11659	299.1275
M331T36			1.329189383	0.103149309	0.338548806	330.95385	36.3295
M275T467	(M-H)-	gamma-L-Glutamyl-L-glutamic acid	5.327203743	0.116111499	1.31586E-15	275.08802	466.671
M302T391_2			0.993960578	0.120434783	0.335318964	302.08053	391.3165
M330T244			4.458316756	0.153574272	0.321890256	330.16628	244.089
M303T368			3.515768345	0.157799606	0.331188729	303.09258	368.3175
M295T380_2			2.175640073	0.179568418	6.43823E-12	295.09257	379.894
M261T470			5.487513057	0.181785578	1.06409E-13	261.07234	469.9
M667T344_1			1.032604948	0.183243761	0.341512514	667.04398	344.124

M385T247			1.411774321	0.188106522	0.332153263	385.15177	247.3035
M159T426			1.72679642	0.20570958	3.80072E-09	159.07684	425.739
M275T142			1.961841525	0.232253626	0.311436088	275.06968	142.158
M753T382			0.956809618	0.246024268	0.317864632	753.45182	382.2385
M363T30			0.588198413	0.249917049	0.325696965	362.94169	30.4555
M276T42_2			0.279230101	0.250405033	0.361405859	276.05333	42.285
M488T402			1.076495919	0.280129454	0.423918632	488.16149	401.8795
M188T283			1.026966965	0.282733343	0.328085332	188.05586	282.874
M419T408			0.629916204	0.284045511	0.316617436	419.22553	408.0485
M204T429			1.624365758	0.286900328	9.81216E-10	204.07009	428.6105
M679T376			1.234232837	0.294893073	0.345268233	679.24696	376.358
M154T429	(M-H)-	L-Histidine	9.499307572	0.314979783	3.50872E-05	154.06162	429.4725
M473T36			4.216610085	0.318841182	0.029975818	473.21699	35.5665
M413T35_2			1.761729088	0.325390852	0.054267652	413.19618	34.814
M203T429			4.987304498	0.327679233	3.03659E-11	203.06678	429.29
M166T168			0.658048948	0.332949135	0.346307244	166.07926	167.6155
M337T35			1.023291254	0.335539766	0.268387893	336.99795	35.0925
M359T469			1.259845169	0.337442791	5.57904E-11	359.04865	469.107
M247T474			1.474078229	0.337777024	1.00151E-09	247.05608	473.6395
M255T39			0.956091689	0.341252701	0.323077044	254.85824	38.7105
M147T142			2.350055086	0.341883932	0.365818594	147.01124	142.46
M730T289			0.691758682	0.343791368	0.368440342	730.45091	289.142
M407T260			1.020624608	0.347840003	0.339705135	407.05351	259.724
M212T301_1			1.051615499	0.348795266	0.329637179	212.05837	300.5375
M772T338			2.490304839	0.356577932	0.013441612	772.0983	338.24
M795T301			0.493045686	0.362793523	0.181413671	795.1438	301.481
M644T347_2			1.080835324	0.371583307	0.334925973	644.25018	347.2425
M104T426			0.744758919	0.372908876	2.71381E-08	104.03462	426.2
M316T380			1.258416405	0.376742295	0.35454816	316.09612	379.874
M464T228			1.499219319	0.377314842	0.006703004	464.32033	227.8065
M201T247_2			1.617652104	0.379358901	0.361788464	201.08732	246.746
M173T248			0.652789678	0.380284605	0.273616783	173.07144	248.0075
M229T474			1.727853116	0.38126907	6.42997E-10	229.04586	473.64
M716T459_1			0.683685331	0.387681245	0.34887627	716.19217	459.1185

M366T357_2			1.388969475	0.396607934	0.003469247	366.12988	357.089
M229T425			2.742232734	0.39719378	0.000102671	229.08226	425.095
M259T151			2.044237196	0.401556448	0.105598126	259.10789	151.337
M602T510			0.454834312	0.405733662	0.307467378	602.451	509.844
M417T472			0.803031855	0.410477344	0.328598061	417.2087	471.53
M185T423_1			2.412960529	0.41179108	0.000753123	185.05622	422.736
M928T228			1.269750829	0.41331929	0.004521811	928.3441	228.3385
M121T172			0.413516112	0.414001274	0.314342057	121.04999	172.144
M243T470_2			3.854700364	0.416898933	1.23396E-11	243.06158	469.891
M411T238_2			0.934578421	0.42848541	0.356695015	411.18708	238.4
M572T224			0.41196967	0.433984875	0.333142847	571.74333	223.5095
M384T411_2			1.467661569	0.437194079	0.31301196	384.16809	410.936
M206T141	(M-H)-	N-Acetyl-L-phenylalanine	1.57064251	0.443302307	0.083851253	206.08133	140.856
M573T204			1.07231863	0.446758604	0.001964403	572.98969	203.727
M501T307			1.939188312	0.446972924	0.216326574	501.1049	307.3515
M263T180			1.021218137	0.448067394	0.000215801	263.13902	179.794
M199T42	(M-H2O-H)-	Primidone	0.780073576	0.448154977	0.280045751	199.08701	42.275
M187T391			1.744930556	0.448223979	2.52148E-06	187.10801	390.567
M644T547			0.597780175	0.448388227	0.031789855	644.41799	547.3115
M945T227			1.335229649	0.449277222	0.027723168	945.34634	227.211
M544T348			0.348714071	0.451908535	0.278489399	544.14832	348.12
M246T350_2			2.054158776	0.453049651	7.7444E-11	246.10868	349.665
M150T228			3.403906657	0.463150977	0.007463013	150.04119	228.028
M201T191			0.630122061	0.463296004	0.311916158	200.95034	191.298
M359T369			0.58960869	0.470995595	0.395192361	359.15593	369.282
M148T129			0.520847853	0.471034679	0.290411795	147.989	129.2815
M289T304			0.973336604	0.471923382	4.10197E-07	289.12706	304.137
M503T232			0.94424945	0.473427014	0.24159586	503.24926	231.8415
M381T28_1			0.590025433	0.474228691	0.043664345	380.7117	28.156
M309T306			1.029131444	0.477994998	4.24507E-07	309.14205	306.343
M371T305			1.021608923	0.482031718	5.84707E-08	371.13019	305.206
M263T381			1.074132554	0.483405521	0.001375108	263.07054	381.303
M260T428			5.257816607	0.48423503	6.67982E-11	260.08843	427.989

M218T291			1.094006993	0.484309954	0.341084965	218.00563	290.879
M131T428			1.291366713	0.487097957	0.044469832	131.04554	428.049
M699T238			0.842969804	0.488736102	0.29999134	699.37344	237.7265
M625T224			0.314781123	0.489082525	0.335176964	624.74607	223.5785
M457T245			3.007515528	0.494045047	0.285187359	457.16123	245.38
M555T232			1.110982507	0.494052757	0.032153028	554.65633	232.4655
M131T84_1			0.306895102	0.494597591	0.233937482	130.98002	84.0605
M603T338			0.592670155	0.494842765	0.261037643	603.17411	337.7065
M157T8			2.609247408	0.494980312	0.008385937	157.12272	8.409
M196T292			0.904953122	0.498369795	3.29146E-06	196.14478	291.867
M245T239			0.493425758	0.49900769	0.334486606	245.08229	238.633
M213T73			0.510781225	0.499583132	0.238699282	212.92734	72.801
M628T296			0.366607081	2.013681716	3.4306E-05	627.95778	295.9325
M482T164			0.596076316	2.016580353	3.0302E-05	482.09004	164.232
M172T43			0.972598364	2.020241702	0.000315591	172.07829	42.877
M539T296			0.961988922	2.024521801	4.04101E-06	538.92703	296.002
M346T414			8.583969047	2.027300126	1.03347E-10	346.05574	413.785
M343T296			2.005079179	2.055230529	1.8443E-07	342.97289	296.179
M357T162			1.450712818	2.081203838	4.38084E-05	357.054	162.422
M350T155			1.945300076	2.093098293	0.000287925	350.14946	155.3675
M300T297			0.584383056	2.098747633	1.81962E-06	299.96693	296.642
M211T414	(M-H2O-H)-	D-Ribulose 5-phosphate	1.844989913	2.100628404	8.7531E-05	211.00064	413.569
M441T296			1.730819662	2.112416003	7.35902E-08	440.94995	295.988
M430T285			2.684454781	2.120119891	0.044854684	430.22896	284.955
M414T159			1.011657089	2.122585649	0.0027805	414.05981	158.878
M705T332			0.530972016	2.123795995	3.51775E-06	704.53715	331.525
M556T232			1.347921945	2.13617022	0.307072709	556.27659	232.3495
M265T53_1			0.702585237	2.13805463	0.381263254	265.00202	52.987
M200T161			1.573928808	2.139552304	8.76341E-05	200.05562	160.915
M325T160			0.779791031	2.145724177	0.00012633	325.06409	160.091
M356T23_2			0.727751348	2.154932661	7.72298E-06	356.02343	23.327
M461T161			0.750875902	2.172318337	0.014986132	461.11117	160.951
M111T297			0.904072653	2.194977487	2.67878E-09	111.01921	296.6425

M419T386_1			0.125545119	2.207744083	0.354626377	418.7339	386.177
M307T524			2.73221216	2.219884123	0.000381237	307.15023	523.803
M101T95			7.381709138	2.227293924	1.53778E-05	101.06055	95.299
M345T297			0.586023888	2.249450075	1.65681E-06	344.97147	296.628
M356T23_1			1.14169394	2.256667794	2.45592E-06	355.52226	23.251
M155T164			0.401581329	2.266388958	0.292671797	155.1182	163.905
M279T161			4.244187435	2.27757677	7.44321E-05	279.03824	160.627
M443T297			0.46701147	2.280564843	0.280671633	443.15225	296.842
M200T217			0.302308795	2.29930246	0.314939401	200.03545	217.276
M120T58			1.1280468	2.375488004	0.365833412	119.97549	58.1355
M381T27			1.564634878	2.376209206	0.056135052	381.23015	26.706
M376T166_2			1.177875243	2.391759732	0.000169438	376.14246	165.6345
M337T96			1.482957157	2.458132159	2.49644E-05	337.18539	96.011
M469T28			0.848197166	2.464216575	0.092843605	469.28245	28.48
M331T81			3.443430728	2.47547313	0.000620113	331.06892	81.4845
M85T34			1.517049845	2.484544693	5.232E-05	84.95048	34.459
M337T26			2.118845877	2.515438899	0.031610308	337.20391	26.27
M372T452			0.631240654	2.560614912	0.248501352	372.06887	451.742
M409T161			2.107996732	2.562445135	0.001399454	409.1239	161.152
M401T164			0.622187385	2.562538484	1.50572E-05	401.08096	163.758
M362T485	(M-H)-	Guanosine 5'-monophosphate (GMP)	1.649846974	2.583803544	0.066113058	362.05002	485.01
M341T410			0.622676087	2.606320823	0.268926487	341.04709	409.7315
M712T23			0.524709416	2.629966406	8.43967E-08	712.05359	23.126
M425T27_2			1.276004604	2.652681914	0.067282404	425.25641	27.487
M581T381			0.311172066	2.653455052	0.374642902	581.34053	380.518
M211T448			1.562451327	2.671935523	1.78248E-07	211.00062	448.4885
M243T161			6.908624043	2.758913055	7.27455E-05	243.062	160.77
M544T436			0.5811529	2.758973729	0.318178655	544.16301	435.8
M267T217	(M-H)-	Inosine	2.672429696	2.770966213	0.003141721	267.07269	216.947
M487T483			0.847258297	2.783939755	0.314041588	486.71179	482.547
M282T582			2.405668894	2.860977345	0.003022685	282.15638	582.19
M459T396			1.749726444	2.882543054	7.99806E-10	459.1344	396.346
M375T165			3.170356623	2.915563151	8.56302E-05	375.13939	165.14

M303T217			0.811518773	3.001540373	0.001284691	303.04886	217.299
M547T405			2.253817634	3.019109745	1.53887E-12	547.11896	404.994
M806T426			0.705590818	3.047662288	0.296923508	806.45269	426.4685
M282T263			2.147831469	3.077491301	0.001363416	282.08352	263.225
M83T35			2.645452656	3.338988009	6.50793E-06	82.95374	34.509
M234T125			0.788147647	3.378026066	0.288706876	233.97334	125.4385
M358T128			0.33488554	3.47904551	0.300506563	358.1246	127.832
M422T196			0.864637933	3.486498888	1.28637E-06	422.23015	196.268
M323T451_1	(M-H)-	Uridine 5'-monophosphate (UMP)	1.91640327	3.536623128	0.002544889	323.0273	451.443
M1126T318			0.848692566	3.553471437	0.311800714	1125.63201	317.9265
M134T169			0.165680782	3.669024115	0.027483169	134.02269	168.5185
M575T510_2			0.781324314	3.744302029	0.336225092	575.44596	509.978
M378T267			2.413235138	3.975941231	5.07326E-07	378.10591	267.138
M421T494			5.463605795	4.018734699	3.33109E-11	421.07491	493.6795
M384T444			0.894741708	4.049367821	2.10773E-05	383.99902	443.664
M249T135			8.066288072	4.20311841	0.35460591	249.05425	134.686
M264T51			0.672092103	4.343915333	0.321104095	263.96341	50.858
M688T254			1.221882936	4.653996516	0.316919977	688.36933	253.5455
M384T272_2			1.258624562	4.676716454	0.34101677	384.19542	271.874
M568T467			0.805910535	4.800372142	0.346425218	567.79856	467.3815
M387T269			1.209877612	5.085469715	0.323264226	387.16667	269.373
M450T200			1.727328706	5.215495974	3.10557E-08	450.26087	199.889
M519T364			1.205023895	5.802643614	0.350299597	519.16712	364.3595
M198T42			1.607079064	5.996308471	0.314350928	198.0222	42.3055
M421T436			1.168490883	6.19241755	2.88963E-12	420.99486	436.17
M384T449			1.777475354	6.206210766	1.90172E-14	384.03104	448.922
M511T236			1.154987666	6.551857728	0.289870302	511.33142	235.895
M274T265			5.042362803	7.104229043	0.000536877	274.09231	265.35
M362T449			11.48453489	7.818984003	1.77598E-15	362.05042	449.326
M202T446			0.907497746	8.242230725	0.333168479	202.05406	446.003
M111T168			0.99415926	9.267217719	0.108418504	111.01927	167.565
M227T239			1.444546361	9.520982932	0.334128195	227.06824	238.659
M374T34_2			2.619449234	12.00475245	0.042610375	374.2441	33.824

M323T423	8.377351773	13.12357069	6.15564E-14	323.02779	422.85
M168T128	1.413010413	13.84992308	0.326281893	168.14681	127.581
